# Amelioration of atherosclerosis in apolipoprotein E-deficient mice by combined RNA interference of lipoprotein-associated phospholipase A_2_ and YKL-40

**DOI:** 10.1371/journal.pone.0202797

**Published:** 2018-08-23

**Authors:** Hui Zhang, Wenping Zhou, Chang Cao, Wenjing Zhang, Gangqiong Liu, Jinying Zhang

**Affiliations:** 1 Department of Cardiology, the First Affiliated Hospital of Zhengzhou University, Zhengzhou, Henan P.R. China; 2 Department of Cardiology, the Third Affiliated Hospital of Zhengzhou University, Zhengzhou, Henan P.R. China; University of Tennessee Health Science Center, UNITED STATES

## Abstract

To test the hypothesis that combined RNA interference (RNAi) of lipoprotein-associated phospholipase A_2_ (Lp-PLA_2_) and YKL-40 is superior to RNAi of Lp-PLA_2_ or YKL-40 alone in ameliorating atherosclerosis. A total of 120 apolipoprotein E-deficient mice (apoE^-/-^ mice) were randomly divided into five groups, including the vehicle alone, scrambled RNAi, Lp-PLA_2_ RNAi, YKL-40 RNAi, and combined Lp-PLA_2_ and YKL-40 RNAi groups. Constrictive collars were used to induce plaque formation. Lp-PLA_2_ RNAi and YKL-40 RNAi viral suspensions were transduced into carotid plaques of the mice. Carotid plaques were harvested for histological analysis four weeks after viral vector transduction. Inflammatory gene expression in the plasma and atherosclerotic plaques was determined by ELISA and real-time PCR. Four weeks after RNAi, the serum concentration and plaque mRNA expression of Lp-PLA_2_ and YKL-40 were remarkably attenuated, leading to reduced inflammatory gene expression. Plaques from the Lp-PLA_2_ or YKL-40 RNAi group showed lower lipid content, higher collagen content, increased fibrous cap thickness, and lower mRNA expressions of MCP-1 and MMP-8 than than those in the vehicle and scramble groups. When compared with the isolated Lp-PLA_2_ or YKL-40 RNAi group, the combined Lp-PLA_2_ and YKL-40 RNAi group exhibited higher collagen content and fibrous cap thickness, and lower lipid content and local inflammation. The beneficial effects of RNAi were independent of the plasma lipoprotein profile. Combined RNAi of Lp-PLA_2_ and YKL-40 is superior to RNAi of Lp-PLA_2_ or YKL-40 alone in ameliorating atherosclerosis.

## Introduction

Atherosclerosis and its clinical complications are the leading causes of death and disability in the western world [1]. It has been increasingly recognized that atherosclerosis is a complex, multifactorial process intertwined with inflammation [2, 3]. Newly described inflammation mediators, such as lipoprotein-associated phospholipase A_2_ (Lp-PLA_2_) and YKL-40, are highly expressed in atherosclerotic plaques and contribute significantly to the progression of atherosclerosis [2]. Lp-PLA_2_ is an enzyme that plays an atherogenic role by hydrolyzing oxidized phospholipids to the proatherogenic lipid mediators lysophosphatidylcholine (LPC) and oxidized non-esterified fatty acid (oxNEFA), both of which contribute to inflammation and render the plaques vulnerable to rupture [4, 5]. Multiple studies have demonstrated a causative role of Lp-PLA_2_ in the development of atherosclerosis [3–5].

YKL-40, a pro-inflammatory cytokine expressed mainly by macrophages, is emerging as a risk factor and a prognostic marker of atherosclerosis [6]. YKL-40 seems especially involved in inflammation and tissue remodeling and is highly up-regulated in atherosclerotic plaques [7, 8]. Plasma YKL-40 is associated with cardiovascular and all-cause mortality [7, 8]. Increased concentrations of both Lp-PLA_2_ and YKL-40 have been reported in patients with atherosclerosis [2–8]. Coronary artery disease is caused by multiple factors. Therefore, it is likely that both Lp-PLA_2_ and YKL-40 may contribute significantly to the formation and progression of atherosclerosis, and we hypothesized that simultaneously down-regulating the expression of Lp-PLA_2_ and YKL-40 may ameliorate atherosclerotic plaques more efficiently than knockdown of Lp-PLA_2_ or YKL-40 alone.

Inflammation inhibition and gene therapy represent a novel approach for addressing atherosclerosis in the future [2, 5]. RNA interference (RNAi) has been shown to be quite efficacious in down-regulating the expression of target genes in a mouse model of atherosclerosis [2, 4]. In the present study, we constructed two lentiviral vectors to down-regulate the expression of Lp-PLA_2_ or YKL-40 following collar-induced atherosclerosis in apolipoprotein E-deficient (apoE^-/-^) mice and tested the hypothesis that knockdown of both YKL-40 and Lp-PLA_2_ together is more effective than knockdown of either alone in ameliorating atherosclerosis in apoE^-/-^ mice.

## Methods

### Cell culture

The mouse RAW264.7 macrophage cell line was purchased from ATCC and routinely cultured in DMEM containing 10% FBS, 100 U/ml streptomycin and 100 μg/ml ampicillin. The cells were cultured to over 90% confluence, and RNAi and scrambled lentiviruses were then used to transduce RAW264.7 cells at a multiplicity of infection (MOI) of 50. The expression of Lp-PLA_2_, YKL-40, MCP-1 and MMP-8 was investigated using quantitative real-time PCR [2, 5]. Negative control (NC) lentiviruses containing scrambled shRNA served as controls.

### Lentiviral vector production

To silence YKL-40 and Lp-PLA_2_ expression, lentiviral shRNA vectors were constructed using 4 different shRNA sequences against YKL-40: 5'-GATGGAACTTTGGGTCTCAAA-3' (YKL-40 Site A)

5'- GCTCCAGTGCTGCTCTGCATA-3' (YKL-40 Site B)

5'- CAATATAAGCAACGATCACAT -3' (YKL-40 Site C)

5'- CCTGACAGATTCAGCAACACT -3' (YKL-40 Site D).

The target sequence (5'-GCAAGCTGGAATTCTCCTTTG-3', Lp-PLA_2_ Site A) for mouse Lp-PLA_2_ mRNA was demonstrated to be effective as previously described [4, 5] and chosen as the target for RNAi in this study. A scrambled NC shRNA lentiviral vector was also constructed using the target sequence 5'-TTCTCCGAACGTGTCACGT-3' (Genepharma, Bioscience, Shanghai, CHINA). Lentiviral vectors were produced in HEK293 cells as previously described [9–10]. Viral titers were 1 × 10^9^ TU (transduction units)/mL as determined by examining green fluorescent protein (GFP). Four lentiviral shRNAs against the YKL-40 shRNA vector were used to transduce the RAW264.7 cells at a MOI of 50. To screen the target for the most effective gene knockdown, transduced RAW264.7 cells were collected for real-time RT-PCR on day 4 following transduction.

### Animals and experimental protocol

Atherosclerotic plaques were elicited in the left common carotid arteries by perivascular collar placement. A total of 120 male apoE^-/-^ mice (C57BL/6 genetic background) were obtained from the Beijing University Animal Research Center and divided into 5 groups according to the method of simple randomization. All animal experiments were approved by the Institutional Committee of Animal Care and Use of Zhengzhou University. ApoE^-/-^ mice received a high-fat diet (0.25% cholesterol and 15% cocoa butter) and underwent constrictive collar placement around the left common carotid artery under anesthesia with an intraperitoneal injection of pentobarbital sodium (30–50 mg/kg) [4,5]. In brief, the common carotid arteries were dissected, and a constrictive silastic collar (inner diameter, 0.30 mm; length, 3 mm) was placed around the left common carotid artery by three circumferential silk ties [4, 5].

Mice were randomly allocated into the vehicle group (n = 24, PBS), scrambled group (n = 24, scrambled shRNA), Lp-PLA_2_ RNAi group (n = 24), YKL-40 RNAi group (n = 24), and combined Lp-PLA_2_ and YKL-40 RNAi group (n = 24, week 1). After six weeks, the carotid collars were removed, and PBS (the vehicle group), scrambled lentivirus (5×10^7^ TU) or lentivirus (5×10^7^ TU, three RNAi groups) was instilled around the plaques of the left common carotid artery. Lp-PLA_2_ RNAi and YKL-40 RNAi viral suspensions were transfected into carotid plaques in the Lp-PLA_2_ RNAi or YKL-40 RNAi groups or in combination in the combined Lp-PLA_2_ and YKL-40 RNAi group. At the end of the experiment (week 10), all mice were sacrificed, and the plaques from the left common carotid arteries were collected for histological analysis.

### Plasma lipids and biological analysis

Plasma was acquired by centrifugation of the blood samples at 1,500 g at 4°C and then stored at -80°C for further analysis. Plasma concentrations of Lp-PLA_2_, YKL-40, MCP-1, MMP-8, total cholesterol (TC), and triglycerides (TG) were measured using quantitative sandwich enzyme immunoassay (commercial ELISA kits) following the manufacturer's recommendation (CoWin Bioscience Co., Ltd.).

### Histological analysis

At the end of week 10, all mice were sacrificed by an anesthetic overdose with intraperitoneal injections of pentobarbital sodium. The left common carotid artery was carefully excised and perfused with 4% formaldehyde, embedded in O. C. T. compound and stored at -20°C [4, 5]. The collar-related plaques formed in the present study were located primarily in the area proximal to the collar [1.2]. Therefore, this section was used for histological analysis. The point of maximal stenosis of each artery was determined by analyzing sections at 100 μm intervals. Serial cryosections (6 μm) were routinely stained with hematoxylin and eosin. The area of maximal plaque size was selected for morphological analysis. Oil red O and Masson’s trichrome staining were used for lipid and collagen visualization, respectively. The intimal area and the medial area were measured using an automated image analysis system (Image-Pro Plus 5. 0; MediaCybernetics, Silver Spring, MD). Lipid- and collagen-positive areas were quantified by a computer-assisted color-gated technique. The percentage of the intimal area that stained positive for lipid and collagen was calculated as previously described [1,4, 5].

### RNA extraction and real-time PCR

Total RNA was extracted from the left common carotid artery following homogenization in Trizol. Reverse transcription was performed following the manufacturer’s protocol (CoWin Bioscience, Beijing, CHINA). SYBR Green RT-PCR was conducted using an ABI Prism 7500 Sequence Detection System (PE Applied Biosystems, Foster City, CA, USA). The specific primers used were as follows: 5'-CCAGAGATTCAGATGTGGAGTT-3' and 5'-TGGCAGAGTTGATAAAGAGGAG-3' for Lp-PLA_2_; 5'- AGGCTTTGCGGTCCTGAT-3' and 5'- CCAGCTGGTGAAGTAGCAGA -3' for YKL-40; 5'-GCTCAGCCAGATGCAGTTAACG-3' and 5'-TCTTGGGGTCAGCACAGACCTC-3' for monocyte chemotactic protein-1 (MCP-1); 5'-GCCTGACTCTGGTGATTTCTTG-3' and 5'-TGTTGATGTCTGCTTCTCCCTG-3' for matrix metalloproteinase-8 (MMP-8); and 5'-GGTGAAGGTCGGTGTGAACG-3' and 5'-CTCGCTCCTGGAAGATGGTG-3' for GAPDH (Jerui-Bioscience, Shanghai, CHINA). The housekeeping gene GAPDH was quantified as an internal control. The relative gene expression levels were calculated by using the 2^-ΔΔCt^ method [5].

### Statistical analysis

Data are presented as the mean values ± standard deviation (SD). If data passed normality test, data were compared with one-way analysis of variance (ANOVA) followed by the Student-Newman-Keuls (SNK) test for post hoc comparisons. All statistical analyses were performed using SPSS 16.0 software (SPSS, Chicago, IL, USA). *P*<0.05 was considered statistically significant.

## Results

### Silencing Lp-PLA_2_ and YKL-40 expression in RAW264.7 cells using lentiviral vectors

**Fig 1:**
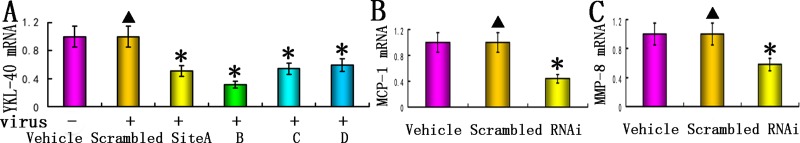
Silencing of YKL-40 in RAW264.7 cells by lentiviral vector-mediated YKL-40 shRNAs. RAW264.7 cells were transduced with 50 MOI of 4 different kinds of shRNA vector, and YKL-40 expression was measured on day 4 by real-time PCR following transduction. GAPDH was used as an internal control. (A) YKL-40 mRNA expression was detected by real-time PCR (n = 8, *P<0.05). (B) RNAi inhibited the expressions of MCP-1 in RAW264.7 cells (n = 8). (C) RNAi inhibited the expression of MMP-8 in RAW264.7 cells (n = 8). No significant difference was found between the vehicle and scrambled groups. Vehicle = vehicle group; Scrambled = scrambled group (negative control). “-” and “+” indicate the absence and presence of lentivirus, respectively. Data are shown as the mean values ± SD obtained from triplicate experiments. *P<0.05 vs. vehicle groups; ^▲^P>0.05 vs. vehicle group.

RAW264.7 cells were transduced with lentiviral vectors expressing four different YKL-40 shRNAs. Ninety-six hours following transduction, the expression of YKL-40 was analyzed using real-time PCR. YKL-40 shRNA B was the most effective and led to an approximately 68.6% reduction in YKL-40 mRNA expression detected by real-time PCR compared to the scrambled group (Fig 1A). YKL-40 shRNAs A, C, and D were less efficient, leading to 49.3%, 55.9%, and 40.8% reductions, respectively, in mRNA expression levels compared to the scrambled group (Fig 1A). Therefore, the YKL-40 shRNA B lentiviral vector was found to be the most effective vector and was selected for further analysis in the present study (Fig 1A). The target sequence (5'-GCAAGCTGGAATTCTCCTTTG-3') against Lp-PLA_2_ mRNA was effective in mice as previously described [2, 4, 5] and selected to knockdown Lp-PLA_2_ in this study. Our previous work has shown that Lp-PLA_2_ RNAi inhibited the expression of MCP-1 and MMP-8 in RAW264.7 cells [4,5], and the expression of MCP-1 and MMP-8 was sharply reduced after YKL-40 RNAi (Fig 1B and 1C). As expected, the vehicle group did not differ from the scrambled group in the mRNA expression of MCP-1 and MMP-8 (Fig 1).

### Silencing Lp-PLA_2_ and YKL-40 expression *in vivo* using a shRNA lentiviral vector

**Fig 2:**
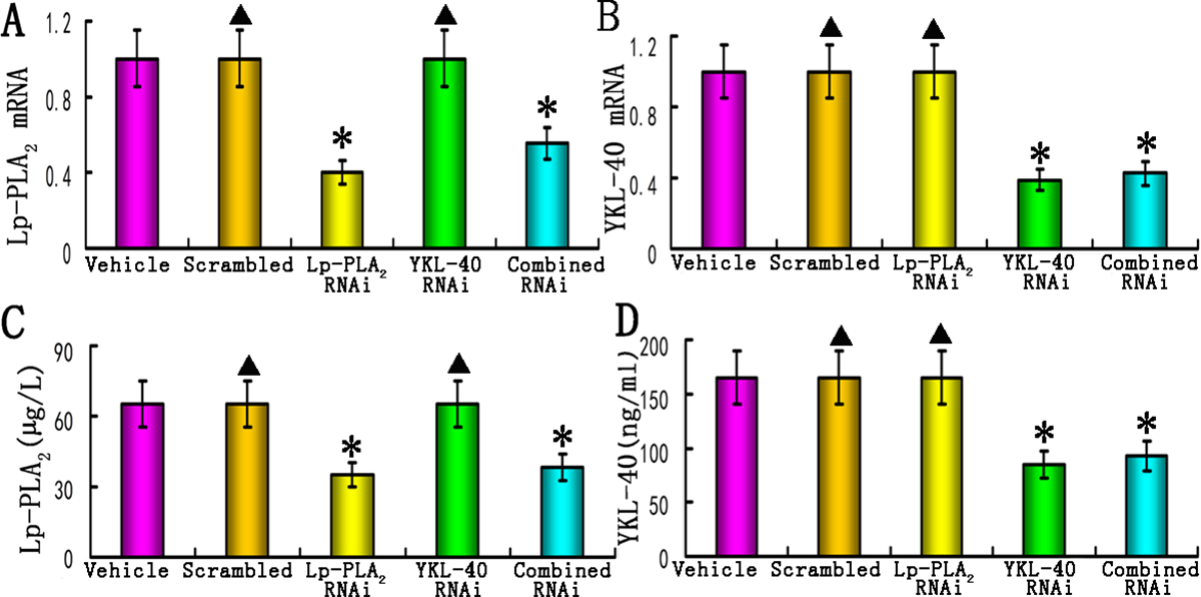
Knockdown of Lp-PLA_2_ and YKL-40 *in vivo*. (A) mRNA expression of Lp-PLA_2_ in the plaques of the vehicle, scrambled, Lp-PLA_2_ RNAi, YKL-40 RNAi, and combined Lp-PLA_2_ and YKL-40 RNAi groups (n = 12 /group); (B) mRNA expression of YKL-40 in the plaques from all groups (n = 12 /group); (C) The plasma concentrations of Lp-PLA_2_ in all groups (n = 24). (D) The plasma concentrations of YKL-40 in all groups (n = 24). Vehicle = vehicle group; scrambled = scrambled group (negative control). Lp-PLA_2_ RNAi = Lp-PLA_2_ RNAi group, YKL-40 RNAi = YKL-40 RNAi group, combined RNAi = combined Lp-PLA_2_ and YKL-40 RNAi group. Data are shown as the mean values ± S.D. *P<0.05 vs. vehicle groups; ^▲^P>0.05 vs. vehicle group.

Our previous studies have demonstrated that local lentivirus shRNA delivery was efficient in inhibiting carotid plaques of apoE^-/-^ mice [2,5]. We examined the mRNA expression of Lp-PLA_2_ and YKL-40 in the carotid plaques and the concentration of Lp-PLA_2_ and YKL-40 in the plasma following lentiviral vector delivery. Compared with the vehicle group, the Lp-PLA_2_ RNAi group together with the combined Lp-PLA_2_ and YKL-40 RNAi group revealed diminished Lp-PLA_2_ mRNA expression by 59.7% and 54.4%, respectively, and reduced plasma concentrations of Lp-PLA_2_ by 55.6% and 51.8%, respectively (Fig 2). In contrast, the Lp-PLA_2_ levels in the vehicle group did not differ from those in the scrambled or YKL-40 RNAi groups (Fig 2).

The YKL-40 mRNA expression level was decreased by 61.2% and 57.5% in the YKL-40 RNAi and combined Lp-PLA_2_ and YKL-40 RNAi groups, respectively, and the plasma concentration of YKL-40 was reduced by 58.7% and 53.9%, respectively, compared to the vehicle group (Fig 2). In contrast, the expression of YKL-40 in the vehicle group did not differ from that in the scrambled or Lp-PLA_2_ RNAi groups (Fig 2).

### Body weight and plasma lipid profiles

We observed no significant difference in body weight among all groups, demonstrating that lentiviral-mediated RNAi did not affect animal growth. Furthermore, TC and TG levels in plasma among all groups were not significantly different, indicating that RNAi did not affect the plasma lipid profile (Table 1).

**Table 1:** Body weight, plasma TC and TG levels among all groups.

	BW (g)	TC (mmol/L)	TG (mmol/L)
Vehicle	27.3 ± 2.5	29.8 ± 3.8	3.2 ± 0.6
Scrambled	26.9 ± 2.4	29.5 ± 3.5	3.0 ± 0.5
Lp-PLA_2_ RNAi	27.1 ± 2.7	30.1 ± 4.0	3.3 ± 0.7
YKL-40 RNAi	27.5 ± 2.8	29.7 ± 3.2	3.5 ± 0.5
Combined RNAi	27.4 ± 2.6	29.3 ± 3.9	3.4 ± 0.6

Data are reported as the mean ± SD of 24 animals. *P* > 0.05 among all groups (one-way ANOVA). BW = body weight; TC = total cholesterol; TG = triglyceride; Vehicle = vehicle group; Scrambled = scrambled group; RNAi = RNA interference group.

### Effects of individual or combined Lp-PLA_2_ and YKL-40 RNAi on the morphology of atherosclerotic plaques

**Fig 3:**
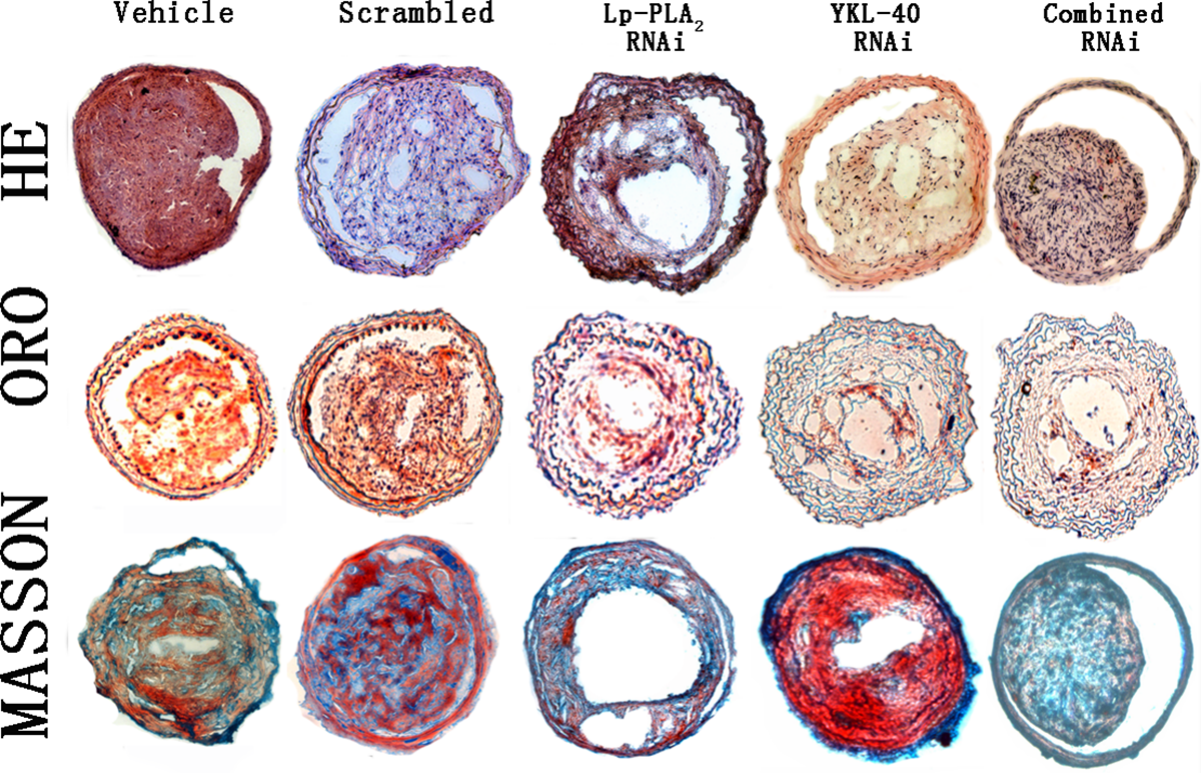
Plaque morphology of the vehicle, scrambled, Lp-PLA_2_ RNAi, YKL-40 RNAi, and combined Lp-PLA_2_ and YKL-40 RNAi groups. Cross-sections of plaques from all groups were stained with HE, ORO and Masson’s trichrome (n = 12). Magnification 200×.

**Fig 4:**
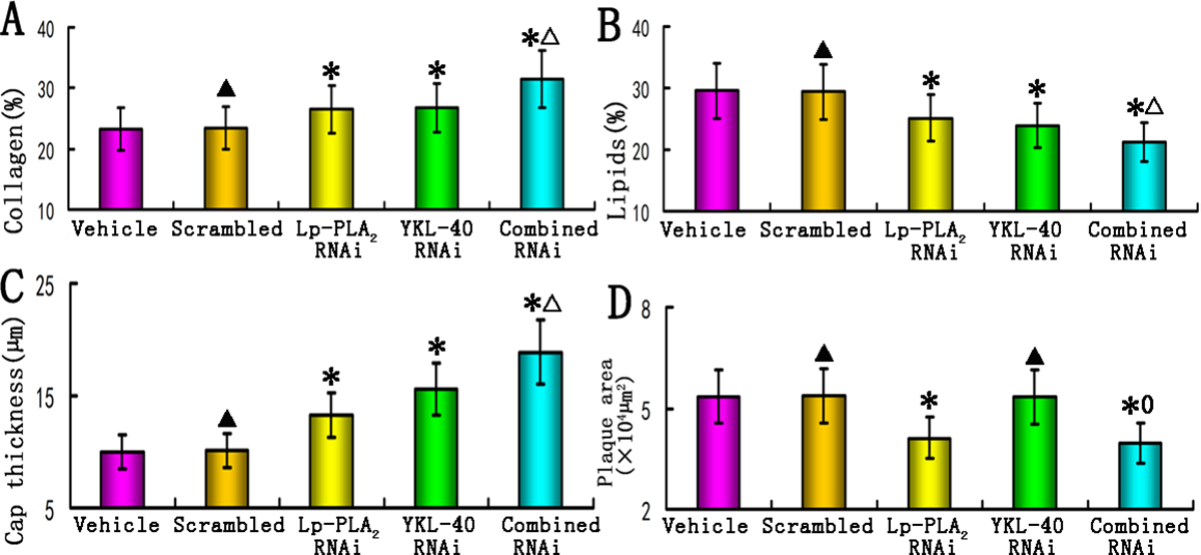
Comparison of plaque morphology. (A) Comparison of relative collagen content in the plaques of the vehicle, scrambled, Lp-PLA_2_ RNAi, YKL-40 RNAi, and combined Lp-PLA_2_ and YKL-40 RNAi groups (n = 12); (B) Comparison of relative lipid content in the plaques from all groups (n = 12). (C) Comparison of fibrous cap thickness in the plaques of all groups (n = 12). (D) Comparison of plaque area in the plaques of all groups (n = 12). *P<0.05 vs. vehicle groups; ^▲^P>0.05 vs. vehicle group; ^△^P<0.05 vs. Lp-PLA_2_ RNAi and YKL-40 RNAi groups. ^0^P>0.05 vs. Lp-PLA_2_ RNAi group.

The plaque area, fibrous cap thickness and relative content of collagen and lipids in carotid plaques were determined by performing immunostaining (Figs 3 and 4). The relative content of collagen in plaques of the vehicle, scrambled, Lp-PLA_2_ RNAi, YKL-40 RNAi and combined Lp-PLA_2_ and YKL-40 RNAi groups was 23.2%, 23.4%, 26.5%, 26.7%, and 31.4%, respectively, and was significantly higher in the three RNAi groups than in the vehicle and scrambled groups (P<0.05), although no significant difference was observed in collagen content between the two isolated RNAi groups (Fig 4A). The combined Lp-PLA_2_ and YKL-40 RNAi group exhibited the highest collagen content, which was significantly higher than that of the other four groups (all *P*<0.05).

The lipid content in plaques from the vehicle, scrambled, Lp-PLA_2_ RNAi, YKL-40 RNAi and combined Lp-PLA_2_ and YKL-40 RNAi groups was 29.6%, 29.4%, 25.1%, 23.9%, and 21.2%, respectively, and was significantly lower in the three RNAi groups than in the vehicle or scrambled groups (Fig 4B). The combined Lp-PLA_2_ and YKL-40 RNAi group exhibited the lowest lipid content, which was significantly lower than that of the other four groups (all P<0.05). In contrast, no significant difference in the lipid or collagen content was found between the vehicle and scrambled groups.

The fibrous cap thickness in plaques from the vehicle, scrambled, Lp-PLA_2_ RNAi, YKL-40 RNAi and combined Lp-PLA_2_ and YKL-40 RNAi groups was 9.98± 0.86 μm, 10.08±0.93 μm, 13.25±1.2 μm, 15.62±1.42 μm and 18.86±1.93 μm, respectively, and was remarkably increased in the three RNAi groups compared with those in the vehicle or scrambled groups (Fig 4C, *P*<0.05). The combined Lp-PLA_2_ and YKL-40 RNAi group exhibited the greatest fibrous cap thickness, which was significantly thicker than that of the other four groups (Fig 4C, all P<0.05).

The plaque area in plaques from the vehicle, scrambled, Lp-PLA_2_ RNAi, YKL-40 RNAi and combined Lp-PLA_2_ and YKL-40 RNAi groups was 5.35×10^4^ μm^2^, 5.37×10^4^ μm^2^, 4.11×10^4^ μm^2^, 5.34×10^4^ μm^2^, and 3.97×10^4^ μm^2^, respectively, and was significantly less in the Lp-PLA_2_ RNAi and combined Lp-PLA_2_ and YKL-40 RNAi groups than in the vehicle, scrambled and YKL-40 RNAi groups (Fig 4D, *P*<0.05). As expected, no significant difference in plaque area or fibrous cap thickness was found between the vehicle and scrambled groups. Interestingly, we also observed that the plaque area for the combined Lp-PLA_2_ and YKL-40 RNAi group was only moderately less than that of the Lp-PLA_2_ RNAi group, and this result was not statistically significant (*P*>0.05) (Fig 4D). These results suggested that Lp-PLA_2_ RNAi or combined RNAi did not differentially affect plaque size. In addition, the plaque area for the YKL-40 RNAi group was not significantly different from that of the vehicle and scrambled groups (P>0.05), indicating that YKL-40 RNAi did not attenuate the atherosclerotic plaque area, although YKL-40 RNAi enhanced the collagen content and fibrous cap thickness of the plaque.

Thus, the RNAi groups showed lower lipid content and higher collagen content than the vehicle and scrambled groups (Fig 4). Fibrous cap thickness was significantly greater in the three RNAi groups than in the vehicle and scrambled groups (*P*<0.01). Taken together, these data indicate that the two individual RNAi groups showed less lipid content and higher collagen content than the vehicle and scrambled groups. Although the three RNAi groups were both effective in attenuating atherosclerotic plaque formation, combined Lp-PLA_2_ and YKL-40 RNAi exhibited higher collagen content and fibrous cap thickness, as well as lower lipid content compared to Lp-PLA_2_ or YKL-40 RNAi alone.

### Effects of RNAi on inflammatory gene expression *in vivo*

**Fig 5:**
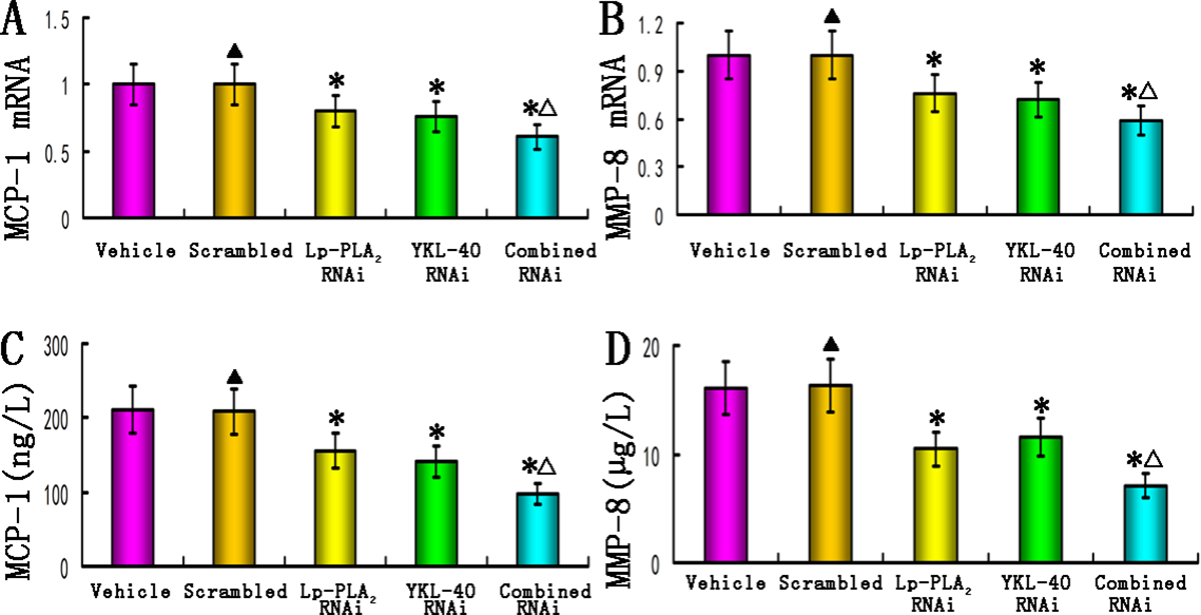
Effects of RNAi on inflammation markers *in vivo*. (A) mRNA expression of MCP-1 in the plaques of the vehicle, scrambled, Lp-PLA_2_ RNAi, YKL-40 RNAi, and combined Lp-PLA_2_ and YKL-40 RNAi groups (n = 12); (B) mRNA expression of MMP-8 in the plaques from all groups (n = 12); (C) The concentrations of MCP-1 in the plasma from all groups (n = 24). (D) The concentrations of MMP-8 in the plasma from all groups (n = 24). *P<0.05 vs. vehicle groups; ^▲^P>0.05 vs. vehicle group; ^△^P<0.05 vs. Lp-PLA_2_ RNAi and YKL-40 RNAi groups.

The mRNA expression of MCP-1 and MMP-8 within the lesion, as well as the concentration of MCP-1 and MMP-8 in the plasma, were significantly lower in the three RNAi groups than in the vehicle or scrambled groups (all *P*<0.05). Furthermore, the combined Lp-PLA_2_ and YKL-40 RNAi group showed significantly lower levels of MCP-1 and MMP-8 compared to the other two single RNAi groups (all *P*<0.05, Fig 5). Additionally, there were no significant differences in the levels of MCP-1 and MMP-8 between the vehicle and scrambled groups (*P*>0.05). Our data demonstrated that the combined Lp-PLA_2_ and YKL-40 RNAi group exhibited enhanced amelioration of plaque inflammation compared to single Lp-PLA_2_ or YKL-40 RNAi, thus providing a potential therapeutic approach for the treatment of atherosclerosis.

## Discussion

In the present study, we assessed the effects of lentiviral-mediated Lp-PLA_2_ and/or YKL-40 RNAi on the progression of atherosclerosis and the associated inflammatory process following collar-induced atherosclerosis in apoE^-/-^ mice. One major finding of the current investigation was that the expression of both Lp-PLA_2_ and YKL-40 in collar-induced atherosclerotic plaques was remarkably attenuated by RNAi. Plaques from the Lp-PLA_2_ and YKL-40 RNAi groups showed lower lipid content, higher collagen content, increased fibrous cap thickness and lower mRNA expression of MCP-1 and MMP-8 than did the vehicle and scrambled groups. Although the three RNAi groups were effective in attenuating atherosclerotic plaque formation, the combined Lp-PLA_2_ and YKL-40 RNAi group exhibited lower lipid content, higher collagen content and fibrous cap thickness and reduced mRNA expression of MCP-1 and MMP-8 compared to Lp-PLA_2_ or YKL-40 RNAi alone, thus providing a potential therapeutic approach for the treatment of atherosclerosis. To the best of our knowledge, this is the first investigation to show that combined RNAi of the Lp-PLA_2_ and YKL-40 genes may enhance the effect of RNAi of the Lp-PLA_2_ or YKL-40 genes alone on atherosclerotic plaques.

Atherosclerosis is a complex, multifactorial process intertwined with inflammation. Inhibiting inflammation factors by gene therapy represents a novel approach to treating atherosclerosis for the future [**2–5**]. Both Lp-PLA_2_ and YKL-40 are mediators of inflammation and are involved in the pathogenesis of atherosclerosis. Lp-PLA_2_ is responsible for the metabolism of oxidized phospholipids to the proatherogenic cytokines LPC and oxNEFA, which trigger significant inflammatory responses and render plaques vulnerable to rupture [4,5]. YKL-40/chitinase-3-like protein-1 is a pro-inflammatory cytokine with roles in injury, repair, angiogenesis and extracellular tissue remodeling, and it is dysregulated in atherosclerosis [11]. YKL-40 is increasingly recognized as a new marker of early inflammation and endothelial dysfunction. Increased concentrations of both markers have been reported in patients with atherosclerosis and those with clinical complications. Coronary artery disease is a multifactorial disease; therefore, it is likely that combined down-regulation of both Lp-PLA_2_ and YKL-40 gene expression would have profound effects on atherosclerotic plaques. In the current work, we constructed two lentiviral vectors to knock down Lp-PLA_2_ and YKL-40 following collar-induced atherosclerosis in apoE^-/-^ mice and elucidate whether selective or combined knockdown of the Lp-PLA_2_ and YKL-40 genes may ameliorate atherosclerotic plaques in apoE^-/-^ mice.

One major finding of the present work was that using lentiviral-mediated RNAi, the expression of Lp-PLA_2_ and YKL-40 can be effectively knocked down in carotid plaques of apoE^-/-^ mice, leading to reduced local inflammatory cytokine expression and plaque lipid content, increased plaque collagen content and fibrous cap thickness. A possible explanation for this beneficial effect might be that RNAi attenuated the expression of inflammatory cytokines in atherosclerotic plaques, as indicated by our cell and *in vivo* experiments that RNAi inhibited the expression of MCP-1, MMP-8, YKL-40 and Lp-PLA_2_ in RAW264.7 cells and *in vivo* [2,5]. Then, the decreased the expression of inflammatory cytokines might favor the reduction of macrophages in the plaque. In the current study, we observed a marked effect of RNAi on the circulating inflammatory markers MCP-1, MMP-8, YKL-40 and Lp-PLA_2_. More importantly, the combined interference of Lp-PLA_2_ and YKL-40 reduces local inflammation more efficiently than the selective interference of Lp-PLA_2_ or YKL-40 alone. These results are in agreement with our previous studies showing that atherosclerosis is a multifactorial process intertwined with inflammation [1,2,5]. In addition, several lines of evidence have demonstrated that high circulating levels of YKL-40 are associated with increased MCP-1, IL-6, and TNF-α levels and macrophage recruitment in plaques [7,12–15]. Furthermore, LPC and oxNEFA, the hydrolyzed end products of Lp-PLA_2_, have been shown to contribute to inflammation and macrophage accumulation in the plaque. Macrophages are the most significant source of Lp-PLA_2_ and YKL-40 in plaques and plasma [2,5]. By virtue of these processes, Lp-PLA_2_ and YKL-40 are involved in a positive feedback loop of inflammation, macrophage recruitment and atherosclerosis.

Macrophages are the main source of pro-inflammatory cytokines, such as MMP-8 and MCP-1, in the plasma and in atherosclerotic plaques. High levels of Lp-PLA_2_, YKL-40, MMP-8, MCP-1 and other pro-inflammatory cytokines may therefore provoke the development of vulnerable plaques. MCP-1 is responsible for the recruitment of macrophages to inflammatory plaques, and MMP-8 is expressed in macrophage-rich plaques, especially the cap shoulder region, which promotes a weakening of the fibrous cap [4,16–17]. MMP-8 possesses proteolytic activity on type I collagen and various matrix and non-matrix proteins [18]. Accumulating evidence indicates that atherosclerotic lesions in MMP-8-deficient mice had increased collagen content [18,19]. Increased concentrations of MCP-1 and MMP-8 are also known to contribute to vascular inflammation, plaque destabilization and thrombosis [18,19].

In the present study, relatively higher levels of Lp-PLA_2_, YKL-40, MMP-8 and MCP-1 were found in the carotid plaques of the vehicle and scrambled groups of mice, and this effect was remarkably reduced by RNAi. This beneficial effect was more pronounced in the combined Lp-PLA_2_ and YKL-40 RNAi group, supporting the idea that atherosclerosis is a complex, multifactorial process intertwined with inflammation [20,21] and that combined interference of Lp-PLA_2_ and YKL-40 reduces local inflammation more effectively than interference of Lp-PLA_2_ or YKL-40 alone; therefore, combined RNAi might play an antiatherogenic and anti-inflammatory role.

We found no significant difference in body weight among all groups, demonstrating that RNAi was safe in these animals. In addition, the plaque area and inflammatory gene expression were not significantly different between the vehicle and scrambled groups, indicating that the beneficial effects observed in the present investigation were not due to non-specific immune stimulation induced by lentiviral transduction. The effects of RNAi were independent of plasma lipoprotein profile, as the TG and TC levels of all groups were not significantly different.

A few limitations of the present study need to be considered; first, we measured only the plasma concentration of Lp-PLA_2_, YKL-40, MMP-8 and MCP-1 at the end of the study, which may not reflect the actual activity of inflammatory cytokines over time. Second, constrictive collar-induced carotid plaques in apoE^-/-^ mice do not fully resemble the process of human plaque rupture and thrombosis formation. However, there is a consensus that the majority of cases of acute clinical manifestation of atherosclerosis are attributed to thrombosis induced by rupture [22]. The propensity to rupture is based on structural characteristics of lesions [22]. Evidences suggest that thicker fibrous cap, relative lower content of lipids, relative higher content of collagen are all indicators of plaque stability [1,2,4,5]. Finally, our data revealed that the plaque area for the combined Lp-PLA_2_ and YKL-40 RNAi group was only moderately lower than that of the Lp-PLA_2_ RNAi group, and this difference was not statistically significant. Further studies are needed to clarify these details.

In summary, our study demonstrated that lentiviral-mediated RNAi was effective in knocking down the expression of Lp-PLA_2_ and YKL-40 in apoE^-/-^ mice, which resulted in reduced expression of inflammatory genes, diminished lipid content, increased collagen content and reduced plaque vulnerability, independent of the plasma lipoprotein profile. In addition, combined interference with Lp-PLA_2_ and YKL-40 is superior to interference with Lp-PLA_2_ or YKL-40 alone in stabilizing atherosclerotic plaques and thus provides a useful approach in ameliorating atherosclerosis.
